# Testostérone and Positive Dimension in Schizophrenia

**DOI:** 10.1192/j.eurpsy.2023.2250

**Published:** 2023-07-19

**Authors:** I. Bouguerra, E. Khelifa, A. Adouni, Y. Sellaouti, H. Ben Ammar, L. Mnif

**Affiliations:** F pyshciatry departement, Razi Hospital, Mannouba, Tunisia

## Abstract

**Introduction:**

Schizophrenia is characterised by a loss of contact with reality due to the presence in its symptomatology of a delusional and/or hallucinatory syndrome, also called positive symptoms and/or a dissociative syndrome, which reflects the negative component of the disease. Few studies suggest a probable link between testosterone and the symptomatic dimension of schizophrenia, but this subject remains poorly documented.

**Objectives:**

The purpose of this study was to describe Testosterone profile in male patients with schizophrenia who are naïve to antipsychotic treatment or have been off it for at least two months and to investigate the relationship between testosterone levels and disease severity.

**Methods:**

This was a descriptive, cross-sectional study of fifty male patients hospitalized for a psychotic relapse who were naïve or discontinuing treatment for at least two months. Patients were assessed using a semi-structured questionnaire and The Positive and Negative Syndrome Scale (PANSS). A blood sample was taken to measure testosterone level.

**Results:**

The age of the patients included ranged from 17 to 65 years, with an average of 36.4±11.51 years. The PANSS score ranged from 50 to 195 with a mean of 116.76 +/- 31.817. Testosterone values ranged from 2.01 to 10.03 ng/ml with a mean of 4.74± 2.01 ng/ml. The majority had normal testosterone levels (94%) ; only 4% had high values and 2% had low values. A positive correlation was found between the positive component of PANSS and elevated testosterone (p=0.011). For the other subscales, no correlation with testosterone levels.

**Conclusions:**

The present study is in favour of a testosterone aggravation of the mostly positive clinical signs of the disease in a significant way. Hormone assays could thus be a specific marker of certain patient profile with a particular evolution.

**Disclosure of Interest:**

None Declared

